# Association between hyperuricemia, gout, urate lowering therapy, and osteoarthritis: A protocol for a systematic review and meta-analysis

**DOI:** 10.1097/MD.0000000000021610

**Published:** 2020-08-14

**Authors:** Junyu Zhu, Yilun Wang, Yuhao Chen, Xiaoxiao Li, Zidan Yang, Hui Li

**Affiliations:** a Department of Orthopaedics, Xiangya Hospital, Central South University; b Hunan Key Laboratory of Joint Degeneration and Injury; c Department of Epidemiology and Health Statistics, Xiangya School of Public Health, Central South University, Changsha, Hunan, China.

**Keywords:** osteoarthritis, serum uric acid, hyperuricemia, urate lowering therapy

## Abstract

**Introduction:**

Osteoarthritis (OA), a chronic and degenerative joint disease characterized by articular cartilage degeneration, sclerosis of subchondral bone, and osteophyte formation, is deemed a leading cause of activity limitation and disability among the elderly people. Serum uric acid (UA) is a terminal metabolite of purine compound, while hyperuricemia (HU) and UA crystals are recognized causes of gout. Several studies have investigated the correlations between HU, gout and OA, but the findings are inconclusive. We are also concerned whether the urate lowering therapy (ULT) can become a potential treatment for OA and intend to undertake this meta-analysis to clarify the related hypotheses.

**Methods:**

Systematic literature search will be conducted on PubMed, Embase, and Web of Science to identify relevant studies up to February 2020 using appropriate search strategies. All citations and abstracts retrieved from literature search will be assessed by two reviewers independently. The Newcastle-Ottawa Scale or the Cochrane risk of bias assessment tool will be used as appropriate to assess the quality and the risk of bias of the included studies. The heterogeneity and the publication bias of the studies will be investigated accordingly.

**Results:**

We propose to undertake this meta-analysis as a feasible approach to clarify the associations between HU, gout or ULT, and OA.

**Discussions:**

This meta-analysis will help to strengthen our knowledge of the pathogenesis of OA and promote the development of preventive or treatment strategies.

**Registration:**

PROSPERO registration number CRD42020168769.

## Abstract 2

Supplemental Digital Content is available in the text

## 1 Introduction

Osteoarthritis (OA) is a chronic and degenerative joint disease characterized by articular cartilage degeneration, sclerosis of subchondral bone, and osteophyte formation.^[1]^ It is a leading cause of activity limitation and disability among the elderly people and can significantly impact the health-related quality of life (QoL).^[2]^ The irreversible joint damage associated with OA may eventually result in the need for total joint replacement (TJR) in order to restore joint function and relieve pain.^[3]^ As a serious public health problem and the most common joint disorder in the global context, OA has imposed a heavy economic burden to the affected patients, their families and the society.^[4,5]^ Several risk factors are believed to be associated with OA, including age, gender, obesity, genetics, etc.^[6]^ However, since the exact pathophysiology of OA is still unclear, there is an urgent need to elaborate the risk factors of OA for the purposes of preventing OA and controlling its progression.

Serum uric acid (UA) is a terminal metabolite of purine compound, which has been indicated as a powerful endogenous antioxidant that can protect cells from oxidative stress-induced damage.^[7,8]^ However, an abnormal increase in serum UA concentration will lead to hyperuricemia (HU), which has a high prevalence in the aged population worldwide.^[9]^ HU has been proven to promote the low-level systemic inflammatory state and has been reported to correlate with a variety of metabolic diseases such as obesity, dyslipidemia, and hypertension.^[10–12]^ Recently, owing to the advance of epidemiology and its basic research, the relationship between OA progression and chronic low-grade systemic inflammation has been further confirmed,^[13]^ which implies a potential association between HU and OA. In contrast, HU and UA crystals are recognized as necessary causes of gout, a common inflammatory arthritis.^[14,15]^ Previous studies have shown that UA crystals could also cause inflammatory reaction in human joints which are always accompanied by the occurrence and development of OA.^[16–20]^ Several studies have examined the correlations between the serum UA level and OA, but the findings are inconclusive. Whether the urate lowering therapy (ULT) has a treatment effect on OA by reducing the serum UA level also remains a question to be addressed.

Meta-analysis has developed rapidly in the recent decade. As a tool with the potential to reveal trends that may be obscurely and paradoxically effective, meta-analysis has been widely used in summarizing previous literature, reaching reliable conclusions, and guiding the establishment of clinical policies and guidelines. Thus, we propose to undertake this meta-analysis as a feasible approach to clarify the associations between HU, gout, or ULT, and OA. To guarantee a comprehensive literature search on OA-related studies in preliminary screening, our outcomes will also include knee replacement (KR), hip replacement (HR), and TJR cases which were caused by OA. These patients may be omitted from incident OA cases, resulting in incomplete data extraction.

## 2 Methods

### 2.1 Study design

The purpose of this meta-analysis is to investigate the associations between HU, gout, or ULT, and OA. This protocol was developed based on the Preferred Reporting Items for Systematic Review and Meta-Analyses Protocols (PRISMA-P),^[21]^ and has been registered in the international prospective register of systematic reviews (PROSPERO) network as suggested by the PRISMA-P.

### 2.2 Eligibility criteria

Observational studies including case-control studies, cross-sectional studies and cohort studies that focus on the associations between HU, gout, or ULT, and OA will be included. Our assessment of OA will target at its prevalence and incidence, as well as the cases undergoing joint replacement. There will be no language restrictions.

### 2.3 Literature search

#### 2.3.1 Information sources

The following electronic databases will be searched: PubMed, Embase, and Web of Science. The search strategy will be formulated based on the goal of identifying all the relevant studies up to March 2020. The references in the retrieved literature will be manually searched in order to scan the related studies and systematic reviews for additional eligible studies.

#### 2.3.2 Search strategy

The search strategy specifies the search keyword terms or medical subject heading terms (MESH) related to the participants of interest, the exposures, the outcomes of interest, and the study type in the databases mentioned above. The full electronic search strategy is illustrated in supplementary file. For each database, search terms will be adjusted appropriately to adapt for different syntax rules.

#### 2.3.3 Data selection

The search results from each individual electronic database will be saved in Endnote. After removing duplicates, the abstracts and titles of the citations will be screened by two investigators independently. The full texts will then be reviewed for inclusion or exclusion according to the pre-specified criteria. Disagreements, if any, will be resolved by discussion, or by consulting to a third investigator if necessary. There will be no restrictions to the publication data and language. The data selection process will be summarized based on the PRISMA flow diagram.

#### 2.3.4 Data extraction

Data will be extracted by two investigators independently using a standardized data recording form. The following research characteristics will be extracted: author, year of publication, study characteristics (i.e., location of the study (country), study period or follow-up period, study design, and sample size), population characteristics (i.e., age and gender), exposure characteristics, and outcome characteristics.

Subsequently, the effect sizes (i.e., mean difference [MD], odds ratio [OR], relative risk [RR], hazard ratio, and confidence intervals [CI]) will be extracted or calculated according to the information from the original study. In case of deficiency of any relevant data, the author(s) of the concerned study will be contacted directly as far as possible. All data entries will also be extracted by the two investigators independently, and disagreements, if any, will be resolved in the same way as mentioned in Data selection.

### 2.4 Assessment of risk of bias

The quality of observational studies will be assessed by the Newcastle-Ottawa Quality Scale (NOS) from three broad perspectives^[22]^:

1. The selection of study groups.
2. The comparability of each group.
3. The ascertainment of exposure for case-control studies or outcome of interest for prospective/retrospective cohort studies.

For cross-sectional studies, a specialized form will be used to adapt to NOS. High methodological quality is defined as the NOS score > 5. Bias assessment will be double-checked by two independent investigators, and any disagreements will be resolved by discussion.

### 2.5 Strategy for data synthesis

The Cochrane Review Manager software (RevMan) and Stata-11.0 statistical software will be used for data assessment. First, the study characteristics will be summarized using baseline tables and narrative texts. The MD, OR, RR, hazard ratio, and the corresponding 95% CIs will be calculated and used to derive the log RR and its standard deviation [SD]. The heterogeneity of the included studies will be assessed by using Cochrane's *Q* test and *I*^*2*^ statistics; statistical homogeneity is defined as *P* value > .05 of the *Q* statistics and *I*^2^ value < 50%. The fixed effects models will be used to consolidate the meta-analysis data if the included studies are proven to be statistically homogeneous; otherwise, the random effects models will be used. *P* value < .05 is considered as statistically significant.

### 2.6 Assessment of publication bias

If there are more than 10 included studies, the publication bias will be assessed by constructing a funnel plot and performing the Egger test. If the funnel plot shows an asymmetric pattern, the existence of publication bias will be confirmed. Any bias will be explained through discussions.

### 2.7 Patient and public involvement

This meta-analysis will be conducted completely based on published studies, so no patients and members of the public will be involved directly.

### 2.8 Ethics and dissemination

As mentioned earlier, our meta-analysis will not involve any patients directly, so no approval from an ethics committee will be required. The results will be published and disseminated through peer-reviewed journals. Our research will not raise any ethical issues.

## 3 Discussions

According to the results of preliminary screening, there are at least 10 studies focusing on the correlations between HU, or gout with or without ULT, and OA. A majority of these studies have reached a consensus that the concerned factors are positively related to OA. Among them, There are six studies indicating that gout is likely to have a positive association with OA,^[23–26]^ or increase the risk of KR, HR, and TJR,^[3,27]^ and one of these studies also pointed out that ULT did not reduce the involved risks.^[3]^ As for HU, four studies suggested a positive association with OA.^[2,28–30]^ In contrast, only one studies reported that there was no association between gout and radiographic OA.^[31]^

For the aged population, OA imposes a great threat to the health of motor system and the QoL.^[2]^ Nowadays, OA has become the most common form of arthritis and has incurred a huge economic burden to the society.^[6]^ Many medical scientists and practitioners are currently working on the treatment of this disease or delay of its progression. Since the pathophysiology of OA had not been fully elucidated yet, comprehensive studies focusing on the etiology and risk factors of OA seem to be necessary.

Unlike earlier views that the progress of OA was only a process of mechanical stress and aging, the present research is more inclined to consider OA as a systemic inflammation.^[13]^ With the continuous intensification of global aging, the prevalence of HU, which is considered an age-related disease, has been increasing all over the world.^[14,32]^ Uric acid, as a product of purine metabolism, has been reported to cause inflammatory reactions in many diseases^[10]^; meanwhile, UA crystals are widely claimed a direct cause of arthritis.^[16]^ Therefore, there may be a certain correlation between the two, but previous studies neither clearly revealed this correlation nor exhibited any obvious trends due to various reasons. Our systematic review and meta-analysis aim to consolidate the available data to investigate the associations between HU, gout, or ULT, and OA. The results will strengthen our knowledge of the pathogenesis of OA and promote the development of preventive or treatment strategies in this field.

## Author contributions

**Conceptualization:** Hui Li

**Data curation:** Junyu Zhu, Yilun Wang, Yuhao Chen, Xiaoxiao Li, Zidan Yang

**Methodology:** Junyu Zhu, Yilun Wang, Yuhao Chen, Xiaoxiao Li, Zidan Yang

**Writing – original draft:** Junyu Zhu and Hui Li

**Writing – review & editing:** Junyu Zhu and Hui Li

## Supplementary Material

Supplemental Digital Content
