# Supplementary material for: Association between hyperuricemia, gout, urate lowering therapy, and osteoarthritis: A protocol for a systematic review and meta-analysis
Source: Medicine (Baltimore). 2020 Aug 14;99(33):e21610. doi: 10.1097/MD.0000000000021610 (PMC7437763; doi:10.1097/MD.0000000000021610)
Supplement: Supplemental Digital Content [file medi-99-e21610-s001.docx]

**Title:** Supplementary file for Association between hyperuricemia, gout, urate lowering therapy, and osteoarthritis: a protocol for a systematic review and meta-analysis

Authors: Junyu Zhu^1^, BM, Yilun Wang^1^, MD, Yuhao Chen^1^, BM, Xiaoxiao Li^2^, MD, Zidan Yang^3^, BM, Hui Li^1^*, PHD

**Authors’ affiliations:**

1. Department of Orthopaedics, Xiangya Hospital, Central South University, Changsha, Hunan, China
2. Hunan Key Laboratory of Joint Degeneration and Injury, Changsha, China
3. Department of Epidemiology and Health Statistics, Xiangya School of Public Health, Central South University, Changsha, Hunan, China

**Email address for all authors:**

Junyu Zhu, junyu_zhu1995@csu.edu.cn

Yilun Wang, yilun_wang@csu.edu.cn

Yuhao Chen, yuhao_chen@csu.edu.cn

Xiaoxiao Li, lixiaoxiao@csu.edu.cn

Zidan Yang, yzd0902@csu.edu.cn

Hui Li^1^, lihui1988@csu.edu.cn

***Correspondence to:**

**Hui Li**, Department of Orthopaedics, Xiangya Hospital, Central South University, Changsha, Hunan, China, E-mail:lihui1988@csu.edu.cn.

**Author’s contributions**

Conceptualization: Hui Li

Data curation: Junyu Zhu, Yilun Wang, Yuhao Chen, Xiaoxiao Li, Zidan Yang

Methodology: Junyu Zhu, Yilun Wang, Yuhao Chen, Xiaoxiao Li, Zidan Yang

Writing – original draft: Junyu Zhu and Hui Li

Writing – review & editing: Junyu Zhu and Hui Li

**1. Pubmed search strategy:**

1. gout[Mesh] OR gout*[Title/Abstract] OR tophus*[Title/Abstract] OR tophi*[Title/Abstract] OR tophaceous*[Title/Abstract]

2. hyperuricemia [Mesh] OR hyperuricemia*[Title/Abstract] OR hyperuricaemia*[Title/Abstract] OR “uric acid”[Mesh] OR “uric acid”[Title/Abstract]

3. “hyperuricemia/therapy”[Mesh] OR “gout Suppressants”[Mesh] OR “uricosuric agents”[Mesh] OR “urate Oxidase”[Mesh] OR allopurinol[Mesh] OR apazone[Mesh] OR benzbromarone[Mesh] OR halofenate[Mesh] OR probenecid[Mesh] OR sulfinpyrazone[Mesh] OR zoxazolamine[Mesh] OR “uric acid lowering therap*”[Title/Abstract] OR “uric acid lowering treatment*”[Title/Abstract] OR “xanthine oxidase inhibit*”[Title/Abstract] OR uricase[Title/Abstract] OR “urate oxidase”[Title/Abstract] OR allopurinol[Title/Abstract] OR apazone[Title/Abstract] OR azapropazone[Title/Abstract] OR benzbromarone[Title/Abstract] OR febuxostat[Title/Abstract] OR halofenate[Title/Abstract] OR pegloticase[Title/Abstract] OR probenecid[Title/Abstract] OR rasburicase[Title/Abstract] OR sulfinpyrazone[Title/Abstract] OR zoxazolamine[Title/Abstract]

4. 1 OR 2 OR 3

5. “osteoarthritis”[Mesh] OR osteoarthriti*[Title/Abstract] OR osteoarthro*[Title/Abstract] OR gonarthriti*[Title/Abstract] OR coxarthriti*[Title/Abstract] OR coxarthro*[Title/Abstract] OR osteo?arthritis[Title/Abstract] OR gonarthro*[Title/Abstract]

6. “arthroplasty, replacement, knee”[Mesh] OR “knee prosthesis”[Mesh] OR “knee/surgery”[Mesh] OR (((knee[Title/Abstract]) OR “knee joint”[Title/Abstract])) AND (((((replace*[Title/Abstract]) OR arthroplast*[Title/Abstract]) OR prosthe*[Title/Abstract]) OR endoprosthe*[Title/Abstract]) OR implant[Title/Abstract]) OR tka[Title/Abstract] OR tkr[Title/Abstract]

7. “arthroplasty, replacement, hip”[Mesh] OR “hip prosthesis”[Mesh] OR “hip/surgery”[Mesh] OR (((hip[Title/Abstract]) OR “hip joint”[Title/Abstract])) AND (((((replace*[Title/Abstract]) OR arthroplast*[Title/Abstract]) OR prosthe*[Title/Abstract]) OR endoprosthe*[Title/Abstract]) OR implant[Title/Abstract]) OR tha[Title/Abstract] OR thr[Title/Abstract]

8. “arthroplasty, replacement”[Mesh] OR “joint prosthesis”[Mesh] OR “joint/surgery”[Mesh] OR (((joint[Title/Abstract]) OR “total joint”[Title/Abstract])) AND (((((replace*[Title/Abstract]) OR arthroplast*[Title/Abstract]) OR prosthe*[Title/Abstract]) OR endoprosthe*[Title/Abstract]) OR implant[Title/Abstract]) OR tjr[Title/Abstract] OR tja[Title/Abstract]

9. 5 OR 6 OR 7 OR 8

10. 4 AND 9

11. Observation[Mesh] OR “Cohort Studies”[Mesh] OR “Longitudinal Studies”[Mesh] OR “Retrospective Studies”[Mesh] OR “Prospective Studies”[Mesh] OR “case-control studies”[Mesh] OR “cross-sectional studies”[Mesh] OR observational[Title/Abstract] OR cohort*[Title/Abstract] OR crosssectional[Title/Abstract] OR crossectional[Title/Abstract] OR cross-sectional[Title/Abstract] OR longitudinal[Title/Abstract] OR case-control[Title/Abstract] OR Epidemiologi*[Title/Abstract] OR retrospective[Title/Abstract] OR prospective[Title/Abstract] OR (cross[Title/Abstract] and sectional[Title/Abstract]) OR longitudinal[Title/Abstract] OR (case[Title/Abstract] and control[Title/Abstract]) OR case-control[Title/Abstract] OR cohort*[Title/Abstract] OR population?base*[Title/Abstract]

12. 10 AND 11

**2. Embase search strategy：**

1. ‘gout’/exp

2. (gout* OR tophus* OR tophi* OR tophaceous*):ti,ab

3. 1 OR 2

4. (‘hyperuricemia’ OR ‘uric acid’)/exp

5. (hyperuricemia* OR hyperuricaemia* OR ‘uric acid’):ti,ab

6. ((‘uric acid’ OR urate) near/3 (elevat* OR high OR raise* OR rise or rising)):ti,ab

7. 4 OR 5 OR 6

8. (‘hyperuricemia/therapy’ OR ‘gout Suppressants’ OR ‘uricosuric agents’ OR ‘urate Oxidase’ OR allopurinol OR apazone OR benzbromarone OR halofenate OR probenecid OR sulfinpyrazone OR zoxazolamine)/exp

9. (‘uric acid lowering therap*’ OR ‘uric acid lowering treatment*’ OR ‘xanthine oxidase inhibit*’ OR uricase OR ‘urate oxidase’ OR allopurinol OR apazone OR azapropazone OR benzbromarone OR febuxostat OR halofenate OR pegloticase OR probenecid OR rasburicase OR sulfinpyrazone OR zoxazolamine):ti,ab

10. 8 OR 9

11. 3 OR 7 OR 10

12. ‘osteoarthritis’/exp

13. (osteoarthriti* OR osteoarthro* OR gonarthriti* OR gonarthro* OR coxarthriti* OR coxarthro*):ti,ab

14. 12 OR 13

15. (‘arthroplasty, replacement, knee’ OR ‘knee prosthesis’ OR ‘knee/surgery’)/exp

16. (knee$ near/5 (arthroplast$ OR prosthe$ OR replac$)):ti,ab

17. ((knee OR ‘knee joint’) AND ((((replace* OR arthroplast*) OR prosthe*) OR endoprosthe*) OR implant) OR tka OR tkr):ti,ab

18. 15 OR 16 OR 17

19. (‘arthroplasty, replacement, hip’ OR ‘hip prosthesis’ OR ‘hip/surgery’)/exp

20. (hip$ near/5 (arthroplast$ OR prosthe$ OR replac$)):ti,ab

21. ((hip OR ‘hip joint’) AND ((((replace* OR arthroplast*) OR prosthe*) OR endoprosthe*) OR implant) OR tha OR thr):ti,ab

22. 19 OR 20 OR 21

23. (‘arthroplasty, replacement, joint’ OR ‘joint prosthesis’ OR ‘joint/surgery’)/exp

24. ((joint OR ‘total joint’) AND ((((replace* OR arthroplast*) OR prosthe*) OR endoprosthe*) OR implant) OR tja OR tjr):ti,ab

25. 23 OR 24

26. 14 OR 18 OR 22 OR 25

27. 11 AND 26

28. (Observation OR ‘Cohort Studies’ OR ‘Longitudinal Studies’ OR ‘Retrospective Studies’ OR ‘Prospective Studies’ OR ‘case-control studies’ OR ‘cross-sectional studies’)/exp

29. (observational OR cohort* OR crosssectional OR crossectional OR cross-sectional OR longitudinal OR case-control OR Epidemiologi* OR retrospective OR prospective OR (cross and sectional) OR longitudinal OR (case and control) OR case-control OR cohort* OR population?base*):ti,ab

30. 28 OR 29

31. 27 AND 30

**3. Web of science search strategy:**

1. TOPIC: (gout)

2. TITLE:(gout* OR tophus* OR tophi* OR tophaceous*)

3. 1 OR 2

4. TOPIC: (hyperuricemia OR “uric acid”)

5. TITLE: (hyperuricemia* OR hyperuricaemia* OR “uric acid”)

6. TITLE:((“uric acid” or urate) NEAR/3 (elevat* or high or raise* or rise or rising))

7. 4 OR 5 OR 6

8. TOPIC:(“hyperuricemia/therapy” OR “gout Suppressants” OR “uricosuric agents” OR “urate Oxidase” OR allopurinol OR apazone OR benzbromarone OR halofenate OR probenecid OR sulfinpyrazone OR zoxazolamine)

9. TITLE:(“uric acid lowering therap*” OR “uric acid lowering treatment*” OR “xanthine oxidase inhibit*” OR uricase OR “urate oxidase” OR allopurinol OR apazone OR azapropazone OR benzbromarone OR febuxostat OR halofenate OR pegloticase OR probenecid OR rasburicase OR sulfinpyrazone OR zoxazolamine)

10. 8 OR 9

11. 3 OR 7 OR 10

12. TOPIC:(“osteoarthritis”)

13. TITLE:(osteoarthriti* OR osteoarthro* OR gonarthriti* OR gonarthro* OR coxarthriti* OR coxarthro*)

14. 12 OR 13

15. TOPIC:(“arthroplasty, replacement, knee” OR “knee prosthesis” OR “knee/surgery”)

16. TITLE:(knee$ NEAR/5 (arthroplast$ OR prosthe$ OR replac$))

17. TITLE:((knee OR “knee joint”) AND ((((replace* OR arthroplast*) OR prosthe*) OR endoprosthe*) OR implant) OR tka OR tkr)

18. 15 OR 16 OR 17

19. TOPIC:(“arthroplasty, replacement, hip” OR “hip prosthesis” OR “hip/surgery”)

20. TITLE:(hip$ NEAR/5 (arthroplast$ OR prosthe$ OR replac$))

21. TITLE:((hip OR “hip joint”) AND ((((replace* OR arthroplast*) OR prosthe*) OR endoprosthe*) OR implant) OR tha OR thr)

22. 19 OR 20 OR 21

23. TOPIC:(“arthroplasty, replacement, joint” OR “joint prosthesis” OR “joint/surgery”)

24. TITLE:((joint OR “total joint”) AND ((((replace* OR arthroplast*) OR prosthe*) OR endoprosthe*) OR implant) OR tja OR tjr)

25. 23 OR 24

26. 14 OR 18 OR 22 OR 25

27. 11 AND 26

28. TOPIC:(Observation OR “Cohort Studies” OR “Longitudinal Studies” OR “Retrospective Studies” OR “Prospective Studies” OR “case-control studies” OR “cross-sectional studies”)

29. TITLE:(observational OR cohort* OR crosssectional OR crossectional OR cross-sectional OR longitudinal OR case-control OR Epidemiologi* OR retrospective OR prospective OR (cross and sectional) OR longitudinal OR (case and control) OR case-control OR cohort* OR population?base*)

30. 28 OR 29

31. 27 AND 30
